# Regional extracellular volume within late gadolinium enhancement-positive myocardium to differentiate cardiac sarcoidosis from myocarditis of other etiology: a cardiovascular magnetic resonance study

**DOI:** 10.1186/s12968-023-00918-z

**Published:** 2023-02-09

**Authors:** Julia Treiber, Dijana Novak, Ulrich Fischer-Rasokat, Jan Sebastian Wolter, Steffen Kriechbaum, Maren Weferling, Beatrice von Jeinsen, Andreas Hain, Andreas J. Rieth, Tamo Siemons, Till Keller, Christian W. Hamm, Andreas Rolf

**Affiliations:** 1 Department of Cardiology, Kerckhoff Heart and Thorax Center, Benekestrasse 2-8, 61231 Bad Nauheim, Germany; 2 Medical Clinic 1, Justus-Liebig-Universität Giessen, Giessen, Germany; 3 German Center for Cardiovascular Research (DZHK), Rhine-Main Partner Site, Frankfurt am Main, Germany

**Keywords:** CMR, Sarcoidosis, Native T1, T2, ECV, Myocarditis

## Abstract

**Background:**

Cardiovascular magnetic resonance (CMR) plays a pivotal role in diagnosing myocardial inflammation. In addition to late gadolinium enhancement (LGE), native T1 and T2 mapping as well as extracellular volume (ECV) are essential tools for tissue characterization. However, the differentiation of cardiac sarcoidosis (CS) from myocarditis of other etiology can be challenging. Positron-emission tomography-computed tomography (PET-CT) regularly shows the highest Fluordesoxyglucose (FDG) uptake in LGE positive regions. It was therefore the aim of this study to investigate, whether native T1, T2, and ECV measurements within LGE regions can improve the differentiation of CS and myocarditis compared with using global native T1, T2, and ECV values alone.

**Methods:**

PET/CT confirmed CS patients and myocarditis patients (both acute and chronic) from a prospective registry were compared with respect to regional native T1, T2, and ECV. Acute and chronic myocarditis were defined based on the 2013 European Society of Cardiology position paper on myocarditis. All parametric measures and ECV were acquired in standard fashion on three short-axis slices according to the ConSept study for global values and within PET-CT positive regions of LGE.

**Results:**

Between 2017 and 2020, 33 patients with CS and 73 chronic and 35 acute myocarditis patients were identified. The mean ECV (± SD) in LGE regions of CS patients was higher than in myocarditis patients (CS vs. acute and chronic, respectively: 0.65 ± 0.12 vs. 0.45 ± 0.13 and 0.47 ± 0.1; p < 0.001). Acute and chronic myocarditis patients had higher global native T1 values (1157 ± 54 ms vs. 1196 ± 63 ms vs. 1215 ± 74 ms; p = 0.001). There was no difference in global T2 and ECV values between CS and acute or chronic myocarditis patients.

**Conclusion:**

This is the first study to show that the calculation of regional ECV within LGE-positive regions may help to differentiate CS from myocarditis. Further studies are warranted to corroborate these findings.

## Background

Cardiac involvement in sarcoidosis frequently leads to malignant arrhythmia and heart failure and is one of the main causes of death in these patients [1, 2]. Rapid diagnosis followed by early, targeted treatment is therefore crucial to improving prognosis. Nevertheless, the diagnosis of cardiac sarcoidosis (CS) can be challenging, and the number of unrecorded cases is high. Endomyocardial biopsy (EMB) is still considered the gold standard of CS diagnosis, but it lacks sensitivity due to its high sampling error of up to 80% [3, 4].

The myocardial late gadolinium enhancement (LGE) pattern in CS patients can be very distinctive, with mass-like patches of LGE in the septal wall and the simultaneous presence of intramural, epicardial, and subendocardial LGE. In more subtle cases, however, differentiation from other types of myocardial inflammation such as viral myocarditis can be challenging.

The advent of new tissue markers such as native T1 and T2 mapping along with measurements of extracellular volume (ECV) have resulted in cardiovascular magnetic resonance (CMR) becoming the cornerstone in CS diagnostics [5, 6]. It allows for identification and quantification of fibrosis or edema by native T1 and ECV measurements and detection of myocardial edema by T2 mapping [7, 8]. However, elevation of global native T1 and T2 values is not specific to CS and is found in acute viral myocarditis as well [9]. Bohnen et al. extended native T1, ECV, and T2 quantification to regional measurements within LGE and found markedly increased values [10].

Histologically, CS differs from viral myocarditis. The hallmark of CS is non-caseating and non-necrotizing granulomas. The predominant T-lymphocyte lineage is CD4+. In inflammation granulomas are replaced by fibrosis similar to that found in myocardial infarction but more evenly distributed over the subendocardial and -epicardial wall [11]. Fibrosis is also found in viral myocarditis but is less extensive and more diffuse [12]. In addition, the highest myocardial 18-fluorodeoxyglucose (^18^FDG) uptake in positron-emission tomography-computed tomography (PET-CT) images is often found in LGE-positive areas of the myocardium.

The aim of the present study was to examine whether including parametric mapping and ECV in LGE-positive regions as proposed by Bohnen et al. can help to distinguish CS from myocarditis.

## Methods

### Patient selection and volunteers

From April 2017 to December 2020 all patients with CMR findings suggestive of CS were included in our registry. CMR was followed by ^18^FDG-PET-CT to confirm diagnosis and to evaluate inflammatory activity [13]. CS was diagnosed in accordance with the updated Japanese diagnostic guidelines in patients with known extracardiac sarcoidosis and two major criteria, or in cases of isolated CS, abnormally high tracer uptake in ^18^FDG-PET-CT and at least three major criteria [14]. Absence of LGE was an exclusion criterion.

Patients with myocarditis were used as a comparison group. Myocarditis was defined according to the criteria proposed by Caforio et al. [15] in the 2013 position paper of the European Society of Cardiology (ESC) Working Group on Myocardial and Pericardial Diseases by:

- Myocarditis-typical tissue characterization by CMR: subepicardial or intramural LGE and elevated native T1 and T2 values
  and at least two of the following features:
- Typical clinical presentation
- Recent-onset arrhythmias in 12-lead electrocardiogram (ECG)
- Elevation of troponin above the 99th percentile [15].

The myocarditis group was further divided into acute myocarditis (symptom duration less than 3 months) and chronic myocarditis (symptom duration more than 3 months). The criterion of acuteness was also adopted from Caforio et al. [15]. Absence of LGE in myocarditis patients was an exclusion criterion.

All patients were extracted from our ongoing prospective clinical native T1 registry that was initiated in April 2017. All patients recruited into the registry were referred for clinically indicated routine CMR, were willing to take part in baseline and follow-up interviews, and consented to provide an additional blood sample for the BioCVI biobank. As of February 2020, 1664 patients had been included. The registry is populated with all-comers patients in a tertiary care center [passage low risk cohort skipped]. Primary indications are ischemia testing, myocarditis, and cardiomyopathy of unknown etiology.

General contraindication for CMR included metallic implants, known gadolinium intolerance, and claustrophobia. Exclusion criteria for this analysis were poor image quality caused by motion artefacts (e.g. atrial fibrillation or ectopic beats) affecting the reconstruction of native T1 and T2 maps, subendocardial or transmural LGE due to ischemic heart disease, and CMR or histological proof of myocardial storage disease.

Coronary artery disease was excluded in all patients of this analysis by invasive angiography but is not generally performed as part of the registry.

All patients and healthy subjects gave written informed consent. The BioCVI registry was approved by the ethics committee of the University of Giessen.

As recommended by the Society for Cardiovascular Magnetic Resonance (SCMR) [16], a reference cohort of 64 healthy subjects was defined who are all employees at our institution. Healthy subjects had no evidence of any cardiac or extracardiac disease, no cardiovascular disease risk factors, and normal body mass index (19–24 kg/m^2^). Healthy subjects underwent native CMR, including cine balanced steady-state free precession (bSSFP) sequences of short axis covering the left ventricle (LV) and right ventricle (RV) and three long-axis (2-chamber, 3-chamber, and 4-chamber) views as well as native T1 and T2 maps in three short-axis views (see below).

### CMR acquisition

All patients were examined on a 3T CMR scanner (Skyra, Siemens Healthineers, Erlangen, Germany) in the head-first, supine position using an 18-channel phased-array surface coil. All CMR protocols contained functional and morphological sequences, and tissue characterization by native T1 and T2 mapping and LGE was carried out in line with the recommendations of the SCMR [17].

### Volumetric measurements

For volumetric measurements bSSFP cine CMR sequences were acquired in standard fashion. Typical parameters for bSSFP-sequences were echo time (TE) 1.38 ms, repetition time (TR) 3.15 ms, flip angle 50°, bandwidth 962 Hz/px, field of view (FOV) 380 mm, voxel size 1.8 × 1.8 × 8.0 mm, slice thickness 8 mm, interslice gap 2 mm, and temporal resolution 30 ms.

### Late gadolinium enhancement

Inversion recovery segmented gradient echo sequences were acquired 10 to 15 min after intravenous injection of Gd-dota (Dotarem®, Guerbet, Villepinte, France) (0.15 mmol/kg bodyweight) in short-axis and 2-, 3-, and 4-chamber long-axis views. Short-axis stacks covered the whole LV with the same center of slice as 5-into-3 planning and cine imaging. The delay between contrast bolus and acquisition was recorded by the technician. Typical parameters were TE 1.97 ms, TR 3.5 ms, flip angle 20°, bandwidth 289 Hz/px, FOV 370 mm, voxel size 1.3 × 1.3 × 8.0 mm, and slice thickness 8 mm with a 2-mm interslice gap.

### T1, T2, and post-contrast T1 mapping

Native T1 and post-contrast T1 maps were generated by using modified Look Locker sequences (MOLLI 3(2)3(2)5), Goethe CVI®, Frankfurt, Germany) before injection of intravenous Gd-dota at the LV base, midventricular, and apical portions following 5 into 3 planning. Typical parameters were TE 1.14 ms, TR 3.1 ms, bandwidth 108 Hz/px, FOV 350 mm, voxel size 1.4 × 1.4 × 8.0 mm, slice thickness 8 mm, adiabatic inversion pulse, 11 inversion times, and ECG-gated antegrade bSSFP single-shot readout with 50° flip angle.

T2 maps were generated before the injection of contrast media using ECG-gated antegrade T2 prep bSSFP sequences during breath hold. Typical parameters were TE 1.34 ms, TR 4.2 ms, flip angle 12°, voxel size 1.8 × 1.8 × 1.8 mm, slice thickness 8 mm, T2 prep with 0, 30, and 55 ms. Three short-axis slices were acquired in identical slice position like T1-maps from base to apex following 5-into-3 planning.

### Postprocessing

**Fig. 1:**
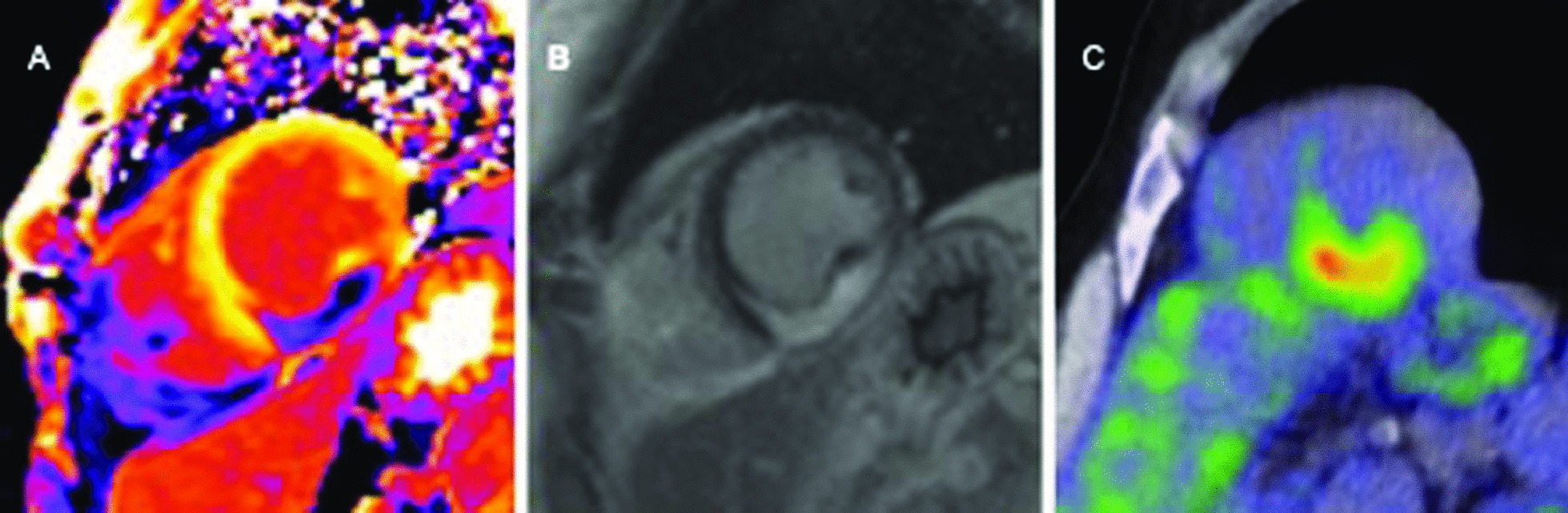
Example for determination of active cardiac sarcoidosis. Imaging findings in one patient with cardiac sarcoidosis (CS) in cardiovascular magnetic resonance (CMR) and 18-fluorodeoxyglucose positron-emission tomography-computed tomography (PET-CT). **A** Shows post contrast T1-map, **B** typical intense intramural Late gadolinium enhancement (LGE) and **C** pathological 18-fluorodeoxyglucose-uptake in PET-CT

Mean native T1 and T2 values were calculated in a region of interest (ROI) at least two voxels wide in midventricular septum for global native T1 and T2 outside LGE areas using ROIs in automatically generated parametric maps (syngovia, Siemens Healthineers). The anatomical shape of the septum was used as reference template. To avoid a partial volume effect the ROI was placed carefully in the center of the septum with distance to the blood pool border as described by the ConSept method [18]. For regional native T1 and T2 values an ROI was drawn in the center of a scar detected by LGE sequences and corresponding pathological ^18^FDG-uptake in PET-CT (Fig. 1). The corresponding LGE image was used as side-by-side reference to assure that regions of interest truly overlapped the areas of LGE. The maps were zoomed large enough to detect single voxel and thereby to avoid voxels outside the LGE areas and border voxels that might carry partial volume signal contamination from blood pool or surrounding tissue.

Tissue characterization by native T1 and T2 mapping in CS and myocarditis patients was compared with that of the control group of 64 healthy subjects to detect deviations from normal.

### Extracellular volume calculation

ECV was calculated by the formula [19]

$$\text{ECV }=\frac{\frac{1}{T1\;postCM\;myocardium} - \frac{1}{T1\;native\;myocardium}}{\frac{1}{T1\;postCM\;blood } - \frac{1}{T1\;native\;blood}}-(1-hematocrit)$$

The hematocrit was measured within 30 min of the CMR by a single antecubital venous blood draw.

### ^18^FDG-PET-CT

After a fasting period of 18 h ^18^FDG was applied as radioactive tracer for differentiation between normal and inflammatory myocardial tissue using the PET-CT technique (Geminin TF 64, Philips Healthcare, Eindhoven, Netherlands). The last meal before the fasting period began was low in carbohydrates and ketogenic. Fifteen minutes before ^18^FDG application the patient was administered 50 I.E./kg bodyweight heparin to suppress the tracer uptake in normal myocardium. After a 18FDG uptake period of 60 to 90 min a Low-Dose CT scan (CTDIvol 2.9 mGy, 5 mm slice thickness) was performed from the crown of the head to the foot immediately before the PET-Scan as basis for the attenuation correction. PET data acquisition was performed in list mode with a measure time of 90 s per bed position, starting subcranial down to the knees. For PET-Reconstruction a standard iterative time of flight algorithm (BLOB-OS-TF) was used with 3 iterations and 33 subsets, 144 × 144 matrix and 4 × 4 × 4 mm^3^ voxel size. Beside attenuation, images were corrected for decay, scatter and random coincidences. Standardized uptake values (SUV) were computed by the scanner software. ^18^FDG PET-CT images were analyzed qualitatively by an experienced radiologist. Myocardial uptake was related to liver uptake. PET-CT was defined as positive by an elevated ^18^FDG uptake within LGE positive regions.

### Statistics

Continuous data are presented as mean ± SD. Categorical data are presented as absolute numbers and percentages. A Kolgomorov Smirnov test was used to test for normality of data. Mean values between groups were compared using ANOVA if data were distributed normally. Post hoc tests between groups were carried out using Scheffé’s test to account for multiple testing. A p-value of less than 0.05 was considered statistically significant. Receiver operating characteristics (ROC) curves were generated to calculate thresholds and areas under the curve (AUC) with 95% confidence interval to test the diagnostic power of CMR tissue markers in differentiating CS from myocarditis. The Youden index was used to define the optimal cut-off to differentiate CS from myocarditis. All tests were performed using Stata software (Stata Corp, College Station, Texas, USA).

## Results

### Basic characteristics

Between April 2017 and February 2020 41 patients were identified with an LGE pattern suggestive of CS in CMR. Five patients had insufficient or missing post-contrast T1 mapping images (three patients with failed motion correction and two missing post-contrast T1-mapping), and three patients showed no ^8^FDG uptake on PET-CT scans. The final CS group comprised 33 patients with CS diagnosed in accordance to the updated 2017 Japanese guidelines [14] who showed both LGE on CMR and pathological regional ^8^FDG uptake. Nine (27%) of these 33 PET-positive patients also underwent EMB. Non-caseating granulomas were not detected in any of the biopsy specimens.

From our registry we identified 73 patients with acute myocarditis and 35 patients with chronic myocarditis but active myocardial inflammation corresponding to the 2013 ESC position paper on myocarditis [15]. Patients with acute myocarditis were 53 (± 18) years old and 21 (29%) were female (Table 1). Patients with chronic myocarditis were 60 (± 14) years old and 14 (40%) were female. The mean age of CS patients was 53 (± 11) years; 14 (42%) were female. There was no difference in N-terminal pro-brain natriuretic peptide (NT-pro-BNP) levels between the groups (p = 0.359). Troponin levels in acute myocarditis were not significantly different from chronic myocarditis. (p = 0.232) (Table 1). Table 2 demonstrates the clinical findings in CS, acute and chronic myocarditis patients (Table 2).

**Table 1:** Baseline characteristics of cardiac sarcoidosis, acute and chronic myocarditis

	Cardiac sarcoidosis n = 33	Acute myocarditis n = 73	Chronic myocarditis n = 35	*p* value
Age, y	54 ± 11	53 ± 18	60 ± 14	0.074
Female	14 (42)	21 (29)	14 (40)	0.296
Arterial hypertension	14 (42)	26 (36)	12 (34)	0.685
Diabetes mellitus	5 (15)	9 (12)	4 (11)	0.959
Chronic kidney disease	6 (18)	8 (11)	4 (11)	0.863
Coronary artery disease	4 (12)	16 (22)	6 (17)	0.177
Supraventricular arrhythmia	11 (33)	12 (16)	7 (20)	0.283
NT-pro-BNP, pg/ml	1423 ± 2192	2468 ± 4792	2873 ± 4270	0.359
Troponin, ng/l	59 ± 133	101 ± 200	34.2 ± 51.4	0.232

Values represent mean (± SD) or n (%)

*NT-pro-BNP* N-terminal pro-B-type natriuretic peptide

**Table 2:** Clinical findings at baseline for cardiac sarcoidosis, acute and chronic myocarditis patients

Diagnostic feature	Sarcoidosis n = 33	Acute myocarditis n = 73	Chronic myocarditis n = 35	p
Heart failure	15 (45)	30 (41)	21 (60)	0.002
Arrhythmia/conduction disturbance	15 (45)	20 (27)	4 (11)	0.002
Chest pain	3 (9)	22 (30)	3 (9)	0.002
Incidental finding/unknown	0	1 (1)	7 (20)	
Pathological 12 lead ECG	27 (84)	56 (77)	18 (51)	0.417
ST/T changes	8 (24)	28 (38)	7 (20)	0.002
Conduction disturbance	18 (55)	12 (16)	7 (20)	0.002
Ectobic beats/tachycardia	1 (3)	16 (22)	4 (11)	0.002
Symptom duration less than 14 days	7 (21)	47 (64)	0	< 0.001

Values present in n (%). Groups are compared by using Χ^2^-test

*ECG* electrocardiogram

The major reason for initial presentation in patients with CS was arrhythmias. While less than half of the acute myocarditis patients had heart failure, the majority of chronic myocarditis patients presented with heart failure (Table 1). The majority of acute myocarditis patients presented with either chest pain or arrhythmias. Almost all patients in all subgroups showed pathological changes in 12-lead ECG (for further information and clinical history see (Table 1), prior infection of the respiratory or gastrointestinal tract were present in 28 patients, 19 in the acute and 9 in the chronic group.

In 42 (39%) of the 108 patients endomyocardial biopsies (EMB) were available; 31 patients were virus positive with predominance of erythorvirus (20), other viruses were HHV6 (6) and Epstein–Barr (EBV) (1). One patient with giant cell myocarditis was detected. Of the virus positive patients 16 belonged to the acute and 15 to the chronic myocarditis group.

### Volumetric measurements

Patients with CS had a higher LVejection fraction (LVEF) and lower LV volumes than patients with myocarditis (Table 3). No difference was observed in volumetric right heart parameters.

**Table 3:** Volumetric measurements and tissue characterization in cardiac sarcoid, acute and chronic myocarditis

	Healthy volunteers	Cardiac sarcoidosis n = 33	Acute myocarditis n = 73	Chronic myocarditis n = 35	p
LVEF, %	63.2 ± 4.7	43.5 ± 14.3	42.8 ± 16.8	45.8 ± 17.5	0.681
LVEDVI, ml/m^2^	86.3 ± 12.2	102.1 ± 29.3	114.2 ± 39.3	106.8 ± 54.4	0.379
LVESVI, ml/m^2^	31.6 ± 5.6	62.1 ± 32.6	65.8 ± 38.5	66.9 ± 52.8	0.894
LV SV, ml		84.3 ± 27.7	89.1 ± 26.1	80.8 ± 26.2	0.315
RVEF, %	51.3 ± 8.3	42.1 ± 11.0	45.2 ± 11.3	47.6 ± 12.7	0.181
RVEDVI, ml/m^2^	88.3 ± 17.8	94.7 ± 26.1	92.9 ± 23.6	85.0 ± 22.9	0.219
RVESVI, ml/m^2^	43.1 ± 12.1	59.6 ± 22.9^b^	51.9 ± 21.4	46.0 ± 21.0^b^	0.051
RV SV, ml		78.4 ± 29.6	82.6 ± 23.9	74.4 ± 25.5	0.3228
ECV global, ms		0.30 ± 0.08	0.34 ± 0.04	0.29 ± 0.07	0.1928
ECV scar, ms		0.65 ± 0.12^a,b^	0.45 ± 0.13^a^	0.47 ± 0.1^b^	** < 0.001**
T1 native global, ms	1121 ± 28	1157 ± 54^a,b^	1196 ± 63^a^	1215 ± 74^b^	**0.001**
T1 post contrast global ms		592 ± 100	615 ± 89	609 ± 71	0.469
T1 native scar, ms		1303 ± 130	1290 ± 66	1289 ± 60	0.747
T1 post contrast scar ms		398 ± 120^a,b^	501 ± 88^a^	514 ± 82^b^	0.00001
T2 global, ms	36.9 ± 2	39.5 ± 3.7	40.2 ± 3.7	40.2 ± 3.5	0.587
T2 scar, ms		44.3 ± 3.7	43.2 ± 5.0	43.1 ± 4	0.644
			Biopsy proven myocarditis only n = 42		
ECV global, ms		0.3 ± 0.08	0.31 ± .09		0.5
ECV scar, ms		0.65 ± 0.12	0.47 ± 0.14		**< 0.001**
T1 global, ms		1157 ± 54	1207 ± 61		**0.001**
T1 scar, ms		1303 ± 130	1293 ± 66		0.7
T2 global, ms		39.5 ± 3.7	39.5 ± 2.9		1
T2 scar, ms		44.3 ± 3.7	41.2 ± 4		**0.04**

Values represent mean ± SD. Mean values were compared using ANOVA. In case of a statistically significant difference (p < 0.05, highlighted by bold values) Scheffé’s test was used as post hoc test. Differing groups are marked by superscript letters

*CS* cardiac sarcoidosis, *LVEDVI* left ventricular end-diastolic volume index, *LVEF* left ventricular ejection fraction, *LVESVI* left ventricular end-systolic volume index, *RVEDVI* right ventricular end-diastolic volume index, *RVEF* right ventricular ejection fraction, *RVESVI* right ventricular end-systolic volume index, *SV* stroke volume

^a^Indicates significance between acute myocarditis and CS

^b^Indicates significance between chronic myocarditis and cardiac sarcoid on the 0.05 level

### LGE

The pattern of LGE in sarcoid patients was patchy with mass like infiltrations of the myocardium especially within the septum. The typical pattern in myocarditis was subepicardial LGE within the lateral wall or intramural LGE of the septum.

### Tissue characterization

Compared with our control group of 64 healthy subjects, global native T1 values were higher for CS (1157 ± 54 ms vs.1121 ± 28 ms; p = 0.002) and acute (1196 ± 63 ms vs. 1121 ± 28 ms; p < 0.001) and chronic myocarditis patients (1215 ± 74 ms vs. 1121 ± 28 ms, p < 0.001) also native T1 values of sarcoid patients were significantly lower than in both acute and chronic myocarditis patients, T2 values were also higher (CS: 39.5 ± 3.7 ms vs. 36.9 ± 2.2 ms; p < 0.001; acute myocarditis: 40.2 ± 3.7 ms vs. 36.9 ± 2.2 ms; p < 0.001; chronic myocarditis 40.2 ± 3.5 ms vs. 36.9 ± 2.2 ms, p < 0.001).

**Fig. 2:**
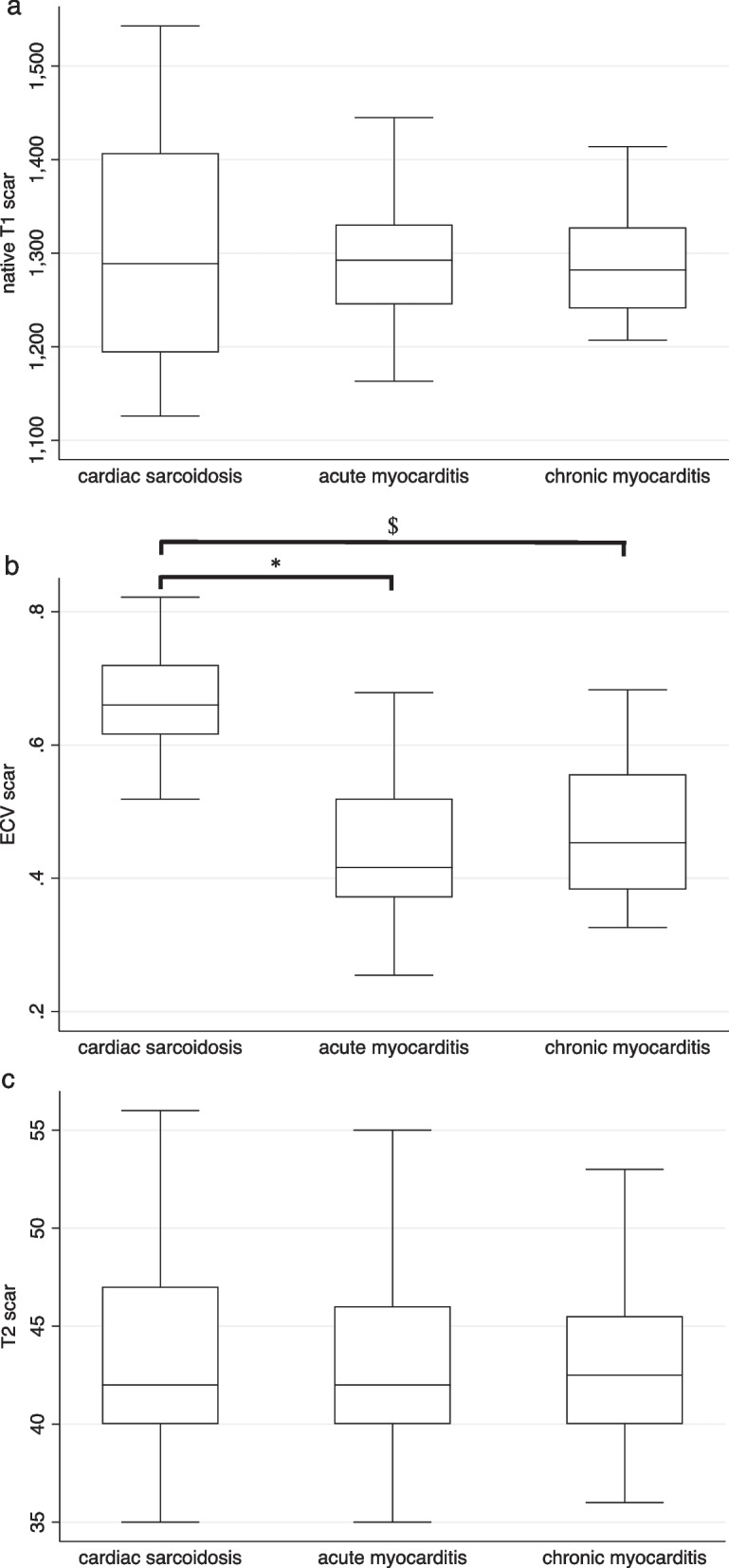

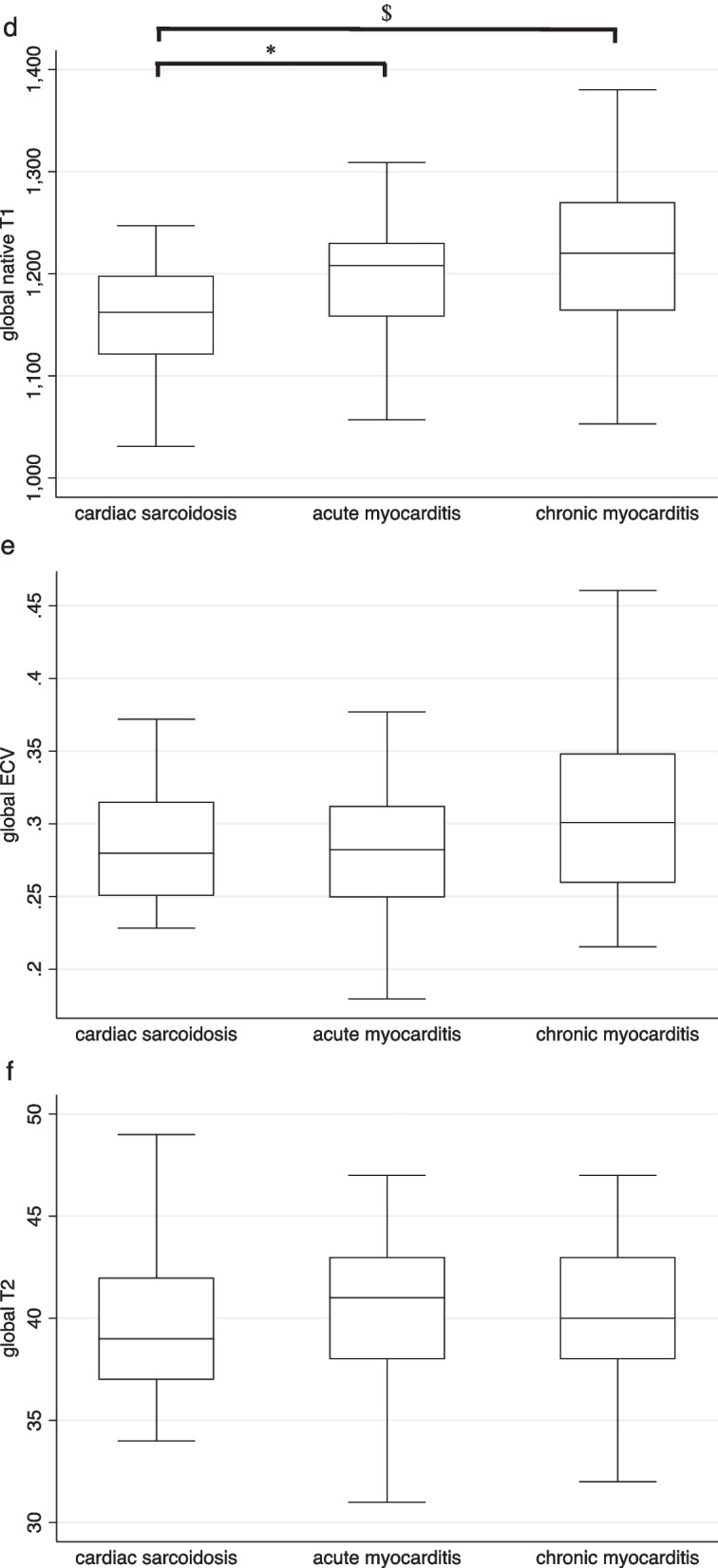
Box-and-whisker plots of tissue characterization in acute and chronic myocarditis and ^18^FDG-PET-CT-positive cardiac sarcoidosis. **a** Native T1 time in scar region; **b** extracellular volume fraction (ECV) in scar region; **c** T2 time in scar region; **d** global native T1 time; **e** global ECV; **f** global T2 time; ^18^FDG-PET-CT: 18-fluorodeoxyglucose positron-emission tomography-computed tomography

CS patients had significantly higher ECV values within LGE regions compared with the myocarditis groups, whereas there was no difference in global ECV, global and regional T2, and regional (LGE) native T1 values between CS and acute or chronic myocarditis patients. CS patients had significantly lower global native T1 values than patients with acute or chronic myocarditis (Table 3, Fig. 2). Confining the analysis to EMB positive patients only yielded similar results (Table 3).

**Fig. 3:**
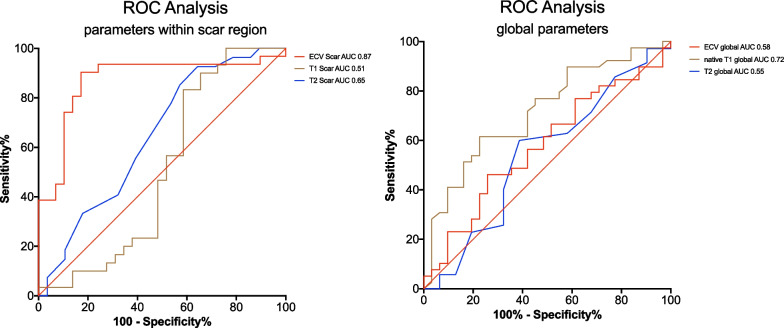
ROC curves for the differentiation of acute and chronic myocarditis and active sarcoidosis by extracellular volume (ECV), T2- and native T1. **a** Regional scar area; **b** global myocardium. ECV in regional scar area shows best AUC with 0.869 (95% CI 0.787–0.952, p < 0.001). *AUC* area under the curve, *CI* confidence interval, *ECV* extracellular volume, *ROC* receiver operator curve

ROC analysis showed the best performance for regional LGE ECV calculation, which yielded an AUC of 0.869 (95% CI 0.787–0.952, p < 0.001) to differentiate between CS and chronic and acute myocarditis. Application of the Youden index showed an ECV within the LGE region of 0.57 as the optimal cut-off for differentiating CS and myocarditis (Fig. 3).

## Discussion

This study was designed to investigate whether parametric measurements and ECV within regions of LGE can differentiate CS from acute and chronic myocarditis. The main findings of our study are:

1. Regional ECV within LGE regions can differentiate CS from active acute or chronic myocarditis. ROC analysis found 0.57 to be the best cut off to differentiate CS and myocarditis.
2. Global native T1, T2, and ECV values in CS patients were elevated compared with healthy controls but not compared with myocarditis patients, regardless of the degree of acuteness. In fact global native T1 was even lower in CS patients.

To the best of our knowledge, this is the first report of a single tissue parameter that shows promise to differentiate these two entities.

The poor prognosis of sarcoidosis patients with cardiac involvement is well documented [1, 20, 21]. Immunosuppressive therapy, in particular using high-dose corticosteroids, is necessary to prevent malignant arrhythmias and progressive heart failure [14]. Consequently, an accurate diagnosis is crucial. However, the variability of clinical presentation and the low diagnostic accuracy of standard diagnostic tools hamper not just the initial diagnosis but also targeted therapy during follow-up [22].

Like many inflammatory diseases, CS is characterized by patchy focal inflammatory areas detectable by LGE in CMR. However, it is not always possible to make a clear distinction between CS and myocardial inflammation of other etiology by the LGE pattern alone. The histological confirmation of non-caseating granuloma is still the diagnostic gold standard for CS, but the high sampling error due to the focal process in CS and the number of possible complications of EMB [23] have led to a shift in the diagnostic paradigm. Accordingly, the 2017 update of the guidelines for diagnosis and treatment of CS in Japan raised the presence of LGE and pathological ^18^FDG-uptake on PET-CT from a minor to a major criterion [14].

CMR and LGE increase the diagnostic yield compared with that of echocardiography, in particular in asymptomatic patients [24, 25]. Consistent with autopsy studies, cardiac involvement can be verified in up to 25% of partly asymptomatic patients with systemic sarcoidosis [26, 27]. LGE also seems to be an independent predictor of adverse events in these patients [28, 29]. In addition, a combination of CMR and ^18^FDG-PET-CT was found to raise the diagnostic accuracy, and in parallel the value of EMB in CS was downgraded [13, 30]. Interestingly, out of 9 EMBs in our cohort, none had non-caseating granulomas.

LGE alone, however, has two drawbacks. Firstly, it only demarcates focal processes and not diffuse diseases, and, secondly, it is not indicative of inflammatory activity. Therefore, native T1 and T2 mapping as well as ECV quantification have attracted more interest and have been reported to be reliable markers of disease activity [31–33]. The use of T1 mapping sequences to quantify fibrosis in myocardium is a well-established technique that is gaining importance in diagnosing and monitoring various cardiomyopathies and their prognosis [34, 35]. Native T1 time and ECV correlate with the portion of fibrosis in histopathological examinations and are therefore an invaluable tool to distinguish between normal and diseased myocardium [36–38]. Native T1 values and ECV are also sensitive to edema. Furthermore, T2 mapping sequences provide the opportunity to identify global and regional edema; here, they are more sensitive than traditional turbo spin echo sequences [39–42]. In combination, native T1, T2, and ECV can help to distinguish fibrosis, edema, or combinations thereof. A few studies showed a difference in native T1 and T2 mapping as well as ECV in global myocardium compared with healthy controls with or without the presence of LGE, even in phenotypically normal patients [32, 33, 43]. In 61 patients with CS, Greulich et al. found that all three parameters—native T1, T2, and ECV—were significantly elevated compared with healthy controls (native T1 994 vs. 960 ms; T2 52 vs. 49 ms; ECV 28 vs. 25%) [44]. Our data are in good agreement with the findings of Greulich et al., and the degree by which parameters were elevated are also on the same order as our results. Puntmann et al. found similar results in 53 patients with biopsy-confirmed CS, matching our findings [32]; however, in contrast to our results global native T1 and T2 values were more markedly increased (T2 54 vs. 45 ms; native T1 1139 vs. 1059 ms at 3 T). Notably, neither of the aforementioned studies included measurements in LGE-positive regions of myocardium. Furthermore, measurements were only compared for CS patients and healthy controls and not with patients with myocardial inflammation of different etiology.

Comparison of the relationship of native T1, T2, and ECV values between myocarditis patients and healthy controls in myocarditis trials shows that relaxation times are similar to those published for CS. For patients with acute myocarditis vs. healthy controls, Lurz et al. reported native T1 values of 1113 vs. 1044 ms and T2 values of 62 vs. 57 ms at 1.5 T [45]. Similarly, Bohnen et al. reported 1113 vs. 1040 ms for native T1 and 62 vs. 55 ms for T2 at 1.5 T [10]. Thus, previous data show that in both CS and myocarditis native T1, T2, and ECV are elevated, but the difference is on the same order, regardless of the underlying disease.

In addition to global native T1 and T2 values to monitor myocarditis, Bohnen et al. described an approach that also incorporates regional native T1 and T2 values within LGE [10]. As global native T1, T2, and ECV values did not allow for a reliable differentiation between CS and myocardial inflammation of other causes, the present study tested the approach of measuring regional relaxation times and ECV to determine whether regional values would differ. The results confirmed this hypothesis, showing a significant difference in regional ECV values that were higher in CS than in both acute and chronic myocarditis. Therefore, the calculation of regional ECV extends the diagnostic output of CMR. This difference was not detected by using native global native T1 alone, in contrast native global native T1 in CS patients was even less elevated than in myocarditis patients. The ECV determined in LGE regions of myocarditis patients is in very good agreement with the findings of the Bohnen et al. paper [10], which underlines the validity of our measurements.

We can only speculate about the histological backdrop of this difference in ECV. native T1 and T2 within LGE were slightly higher in CS patients than in myocarditis patients but not significantly so. Thus, the higher ECV can be only partially attributed to edema. While lymphocytic infiltration and fibrosis in myocarditis are diffuse and evenly distributed over regions of myocardium, there is a more severe disruption of the healthy myocardial architecture with possibly more extracellular space taken up by granulomas and replacement fibrosis in sarcoidosis. In that respect LGE in CS likely resembles tissue replacement seen in myocardial infarction, which shows similar ECVs as we have found here, in fact the ECVs reported here are even slightly larger than in myocardial infarction. Tissue infiltration by granulomas, lymphocytes, fibroblasts and replacement fibrosis also seems to be a more focal process than viral or post viral myocarditis. The more focal nature of sarcoidosis is also reflected by lower global native T1 of CS patients compared to both acute and chronic myocarditis [11].

These results also reflect what is observed in PET-CT, where the highest ^18^FDG uptake is usually found in those area of the myocardium that also show LGE. Our findings highlight that, in addition to the traditional use of CMR, the calculation of ECV in regional scarring could be a useful parameter in the diagnostic approach for CS. The calculation of ECV in regional LGE can provide valuable additional information and may be helpful for the differentiation not only between diseased and healthy myocardium but also between other myocardial inflammatory diseases and active or inactive LGE foci if PET/CT is not available.

### Limitations

There are limitations to our study. The number of CS patients was comparatively low, although the orders of native T1 and T2 values measured are in good agreement with those of the Greulich and Puntmann studies [32, 44], and regionally measured values within LGE of myocarditis patients match data from Bohnen et al. [10]. A further limitation is the retrospective character of our analysis. In addition, mapping sequences were generated in three slices, raising the possibility that scar regions could be missed in these images in future studies and in different patients. Finally, there was no histological confirmation of CS by EMB in our cohort, although all patients of the CS cohort had both positive CMR and PET-CT findings in accordance with the Heart Rhythm Society consensus paper [21]. A multicenter study would be desirable to validate our findings in a larger cohort.

### Conclusion and perspectives

Our study is the first to show that regional ECV within regions of LGE helps to differentiate CS from acute and chronic myocarditis. Therefore, native T1 and T2 mapping sequences and the calculation of ECV should be implemented in a standard CMR acquisition protocol for a more in-depth assessment and understanding of focal inflammatory processes. Prospective studies are needed to confirm theses exploratory findings.

## Data Availability

Access to the data can be granted by request to the authors.

## Abbreviations

^18^FDG: 18-Fluorodeoxyglucose
AUC: Area under the curve
bSSFP: Balanced steady-state free precession
CMR: Cardiovascular magnetic resonance
CS: Cardiac sarcoidosis
ECG: Electrocardiogram
ECV: Extracellular volume
EDVI: End-diastolic volume index
EMB: Endomyocardial biopsy
ESVI: End-systolic volume index
FOV: Field of view
HRS: Heart Rhythm Society
IQR: Interquartile range
LGE: Late gadolinium enhancement
LV: Left ventricle/left ventricular
LVEDVI: Left ventricular end-diastolic volume index
LVEF: Left ventricular ejection fraction
LVESVI: Left ventricular end-systolic volume index
NT-pro-BNP: N-terminal pro-B-type natriuretic peptide
PET-CT: Positron-emission tomography-computed tomography
ROC: Receiver operating characteristics
RV: Right ventricle/right ventricular
RVEDVI: Right ventricular end-diastolic volume index
RVEF: Right ventricular ejection fraction
RVESVI: Right ventricular end-systolic volume index
SCMR: Society of Cardiovascular Magnetic Resonance
SV: Stroke volume
TE: Echo time
TR: Repetition time
